# Supplementary material for: Cloud BioLinux: pre-configured and on-demand bioinformatics computing for the genomics community
Source: BMC Bioinformatics. 2012 Mar 19;13:42. doi: 10.1186/1471-2105-13-42 (PMC3372431; doi:10.1186/1471-2105-13-42)
Supplement: Additional file 1 — Supplementary 1 Cloud BioLinux software documentation in the form of a mini, self-contained website. Users need to download and uncompress the .zip file, and open through a web browser the "index.html" file available on the main directory. (ZIP 1823 kb). [file 1471-2105-13-42-S1.ZIP › Cloud-BioLinux-Package-Documentation/docs/nthseqset.html]

Bio-Linux Software Documentation Pages

Back to search form

## nthseqset

|  |  |
| --- | --- |
| Name | nthseqset |
| Description | **nthseqset** writes a single sequence alignment (set) from an input stream of sequence sets into file. The sequence set is specified by number, where the number reprsents the order the sequences appear in the input file.    **nthseqset -h** for basic programme options    **nthseqset -h -v** for further programme options     **tfm nthseqset** for full program informations |
| Homepage | http://emboss.sourceforge.net |
| Remote Documentation | http://emboss.sourceforge.net/apps/release/6.3/emboss/apps/nthseqset.html |

EMBOSS documentation for nthseqset
